# A Case of Terbinafine-Resistant Tinea Cruris Caused by *Trichophyton tonsurans*

**DOI:** 10.1155/2021/9611072

**Published:** 2021-11-29

**Authors:** Alireza Firooz, Ensieh Lotfali, Mahsa Fattahi, Maryam Fattahi, Akram Miramin Mohammadi, Mahshid Shahrzad Kavkani

**Affiliations:** ^1^Center for Research and Training in Skin Diseases and Leprosy, Tehran University of Medical Sciences, Tehran, Iran; ^2^Department of Medical Parasitology and Mycology, School of Medicine, Shahid Beheshti University of Medical Sciences, Tehran, Iran; ^3^Emergency Medicine Management Research Center, Iran University of Medical Sciences, Tehran, Iran; ^4^Department of Microbial Biotechnology, Islamic Azad University, Damghan, Iran

## Abstract

A 26-year-old male patient referred to our center with a history of extremely itchy crusted skin lesions in his groins for one year. Moreover, his friend, a 25-year-old male, also developed similar lesions in the groin after using the shared pool, whose condition also did not improve with similar treatment. A regular mycology test (direct and culture test) was performed, as well as molecular examination. The antifungal susceptibility assay to terbinafine, itraconazole, posaconazole, fluconazole, and voriconazole was conducted according to the Clinical and Laboratory Standards Institute M38 third ed. The sequencing study identified *T. tonsurans* as the causative organism in both patients. The abovementioned organism isolated from both patients displayed resistance against terbinafine and fluconazole (MIC ≥ 4 *µ*g/ml and MIC ≥ 8 *µ*g/ml, respectively). Moreover, the susceptibility of both subjects to posaconazole (0.313 *µ*g/ml), voriconazole (0.25–0.0625 *µ*g/ml), and (1 *µ*g/ml) itraconazole increased. The present report aimed to emphasize the increase in antifungal resistance and a demand for antifungal stewardship, to control this public health threat.

## 1. Introduction

Dermatophytosis is known as the most common type of skin disease in the world which is caused by dermathophytes. It is approximated that dermatophytosis affects more than 20–25% of the world's population [1, 2].

## 2. Case Presentation

**Figure 1:**
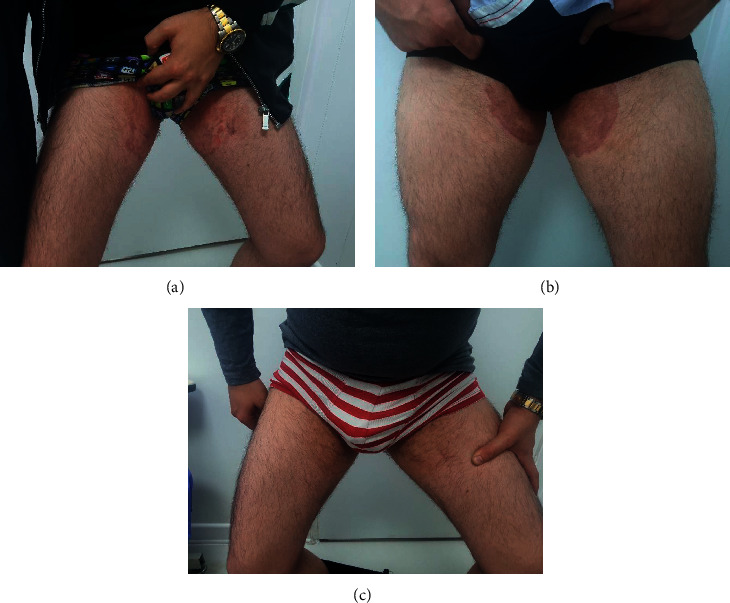
(a, b) Two patients with groin dermatophytosis which showed resistance to terbinafine; (c) sample of the same patients after treatment with voriconazole.

A 26-year-old male patient referred to our center with a history of extremely itchy crusted skin lesions in his groins for one year (shown in Figure 1(a)). Terbinafine tablet (250 mg/day) for 4 weeks and then fluconazole capsule (150 mg/day) for 6 weeks, clotrimazole cream, and antifungal shampoos were prescribed for him. Nevertheless, they were not able to improve his situation and his symptoms worsened. Moreover, his friend, a 25-year-old male, also developed similar lesions in the groin after using the shared pool, whose condition also did not improve with similar treatment (shown in Figure 1(b)). A regular mycology test (direct and culture test) was performed, and dermatophytosis caused by *Trichophyton* was confirmed. We isolated genomic DNA from a seven-day colony cultivated on Mycosel Agar (Merck, Germany) at 28°C using a genomic DNA extraction kit (Roche Life Science, Germany). Then, we amplified the ITS1/ITS2 area and sequenced the PCR products. Afterward, we made a comparison between the sequences and the reference data of the GenBank database via the BLAST program (http://www.ncbi.nlm.nih.gov/BLAST).

We also performed an antifungal susceptibility test to help us choose a proper treatment. We bought the antifungals from Sigma-Aldrich, USA. The *in vitro* activities of antifungal agents, including terbinafine, itraconazole, posaconazole, fluconazole, and voriconazole, were assessed by the broth microdilution protocol that was recommended by the Clinical and Laboratory Standards Institute M38 third ed. We selected *Candida parapsilosis* ATCC 22019 as the quality control strain in all the runs.

The sequencing study identified *T. tonsurans* as the causative organism in both patients and deposited in the GenBank OK479141. The abovementioned organism isolated from both patients displayed resistance against terbinafine and fluconazole (MIC ≥ 4 *µ*g/ml, MIC ≥ 8 *µ*g/ml, respectively). Moreover, the susceptibility of both subjects to posaconazole (0.313 *µ*g/ml), voriconazole (0.25–0.0625 *µ*g/ml), and itraconazole (1 *µ*g/ml) increased.

They were treated for 3 months with itraconazole 100 mg for 3 months, but did not show favorable response. So, they were treated with voriconazole tablets 200 mg/day for two months which cleared the lesions (shown in Figure 1(c)). Then, the patients continued maintenance therapy with itraconazole (100 mg/day) for a month. They were followed up for 3 months and did not show any sign of recurrence. The patients were also given topical clotrimazole cream with both voriconazole and itraconazole.

## 3. Discussion

Treatment-resistant dermatophytosis is a public health issue that has recently become common. Nevertheless, the resistance of *T. tonsurans* to terbinafine is rare. The first case of resistant *T. tonsurans* was discovered *in vitro* [3]. Chen et al. reported a 62-year-old man who had Darier disease complicated by terbinafine-resistant *T. rubrum.* He was treated successfully with itraconazole [4]. We did not find any report of resistant cases to both terbinafine and fluconazole in the literature.

Long exposure to subinhibitory concentrations of antifungals is mentioned as a possible reason for drug resistance [5]. Resistance of *T. tonsurans* against terbinafine could be due to the changes in the squalene epoxidase gene [6, 7] that was implied in patients with *Trichophyton interdigitale* as well. It must be noted that the treatment of resistant *T. tonsurans* is difficult. Considering that these cases showed resistance to terbinafine and fluconazole, we had to prescribe voriconazole, which is an antifungal typically used for the treatment of severe fungal infections in immunocompromised cases. The symptoms and rash disappeared two months after the daily consumption of voriconazole 200 mg.

## 4. Conclusions

The present report aimed to emphasize the increase in antifungal resistance and a demand for antifungal stewardship, to control this public health threat.

## Data Availability

The data used to support the findings of this study are included within the article.

## References

[1] Havlickova B., Czaika V. A., Friedrich M. (2008). Epidemiological trends in skin mycoses worldwide. *Mycoses*.

[2] Hayette M.-P., Sacheli R. (2015). Dermatophytosis, trends in epidemiology and diagnostic approach. *Current Fungal Infection Reports*.

[3] Salehi Z., Shams-Ghahfarokhi M., Razzaghi-Abyaneh M. (2018). Antifungal drug susceptibility profile of clinically important dermatophytes and determination of point mutations in terbinafine-resistant isolates. *European Journal of Clinical Microbiology & Infectious Diseases*.

[4] Chen E., Ghannoum M., Elewski B. E. (2021). Treatment‐resistant tinea corporis, a potential public health issue. *British Journal of Dermatology*.

[5] Fattahi A. (2020). Multidrug‐resistant Trichophyton mentagrophytes genotype VIII in an Iranian family with generalized dermatophytosis: report of four cases and review of literature. *International Journal of Dermatology*.

[6] Hsieh A., Quenan S., Riat A., Toutous-Trellu L., Fontao L. (2019). A new mutation in the SQLE gene of Trichophyton mentagrophytes associated to terbinafine resistance in a couple with disseminated tinea corporis. *Journal de Mycologie Médicale*.

[7] Rudramurthy S. M., Shankarnarayan S. A, Dogra S (2018). Mutation in the squalene epoxidase gene of Trichophyton interdigitale and Trichophyton rubrum associated with allylamine resistance. *Antimicrobial Agents and Chemotherapy*.

